# Development of a dual-arm rapid grape-harvesting robot for horizontal trellis cultivation

**DOI:** 10.3389/fpls.2022.881904

**Published:** 2022-09-20

**Authors:** Yingxing Jiang, Jizhan Liu, Jie Wang, Wuhao Li, Yun Peng, Haiyong Shan

**Affiliations:** Key Laboratory of Modern Agricultural Equipment and Technology, Jiangsu University, Zhenjiang, China

**Keywords:** grape, standard trellis, sequential mirroring, depth threshold segmentation, one-eye and dual-hands visual servo

## Abstract

It is extremely necessary to achieve the rapid harvesting of table grapes planted with a standard trellis in the grape industry. The design and experimental analysis of a dual-arm high-speed grape-harvesting robot were carried out to address the limitations of low picking efficiency and high grape breakage rate of multijoint robotic arms. Based on the characteristics of the harvesting environment, such as the small gap between grape clusters, standard trellis, and vertical suspension of clusters, the configuration of the dual-arm harvesting robot is reasonably designed and analyzed, and the overall configuration of the machine and the installation position of key components are derived. Robotic arm and camera view analysis of the workspace harvesting process was performed using MATLAB, and it can be concluded that the structural design of this robot meets the grape harvesting requirements with a standard trellis. To improve the harvesting efficiency, some key high-speed harvesting technologies were adopted, such as the harvesting sequence decision based on the “sequential mirroring method” of grape cluster depth information, “one-eye and dual-arm” high-speed visual servo, dual arm action sequence decision, and optimization of the “visual end effector” large tolerance combination in a natural environment. The indoor accuracy experiment shows that when the degree of obscuration of grape clusters by leaves increases, the vision algorithm based on the geometric contours of grape clusters can still meet the demands of harvesting tasks. The motion positioning average errors of the left and right robotic arms were (*X*: 2.885 mm, *Y*: 3.972 mm, *Z*: 2.715 mm) and (*X*: 2.471 mm, *Y*: 3.289 mm, *Z*: 3.775 mm), respectively, and the average dual-arm harvesting time in one grape cluster was 8.45 s. The field performance test verifies that the average harvesting cycle of the robot with both arms reached 9 s/bunch, and the success rate of bunch identification and harvesting success rate reached 88 and 83%, respectively, which were significantly better than those of existing harvesting robots worldwide.

## Introduction

Grapes are soft-skinned, juicy berries that occupy an important position in the world of fruit production. In 2020, the total area of vineyard cultivation worldwide was 7.3 million hectares, there were 50 million tons of grapes produced in the world, and China produced approximately 20 million tons, creating substantial economic value for people worldwide (Organisation internationale de la vigne et du vin [OIV], 2020; Isrigova et al., 2021). Due to growing labor shortages, the need for harvesting robots for fresh grapes has become increasingly urgent.

Trellis grapes are mainly used for fresh fruit consumption and are extremely difficult to harvest because of the need to ensure the integrity of grape clusters and soundness of grapes for transportation and marketing requirements (Ozkan et al., 2007; Jianying et al., 2014; Zhu et al., 2014). Traditional trellis grape harvesting operations rely mainly on manual work performed by two hands to finish working together, one hand to support and the other to cut grape stems, to complete one grape harvesting process (Piazzolla et al., 2016; Wang et al., 2017). This harvesting model is both inefficient and has high labor costs and will not meet the rapid harvesting standard of the future grape industry. Grape trellis configurations are mostly horizontal in Asia, the planting height is as high as 2 m, and the harvesting point of grape stems is usually 1.8 m above the ground. Traditional single-arm harvesting robots have deficiencies such as long harvesting cycles, poor moving flexibility, and inaccurate fruit harvesting accuracy, and they cannot meet the requirements of grape harvesting in standard trellises (Possingham, 2006; Suvoéarev et al., 2013; Williams and Fidelibus, 2016). Therefore, a highly efficient harvesting robot must be designed for standard trellis grapes to address the embarrassing gap of a lack of reliable harvesting machines in the grape-growing industry.

At present, researchers worldwide are still in the exploratory stage of research on harvesting machinery for grapes on trellises, and their research methods mainly revolve around visual positioning identification of grape clusters and the design of end-effector configurations (Luo et al., 2016; Liu et al., 2019; Tang et al., 2020; Kalampokas et al., 2021; Majeed et al., 2021; Peng et al., 2021). Facing the growth characteristics of different types of fruits and vegetables, researchers have developed multiple types of picking equipment. Mehta et al. (2014) proposed a cooperative vision servo controller for autonomous harvesting to adjust the position of the end effector according to the real-time position of fruit and, to a certain extent, to weaken the interference of the complex environment in the harvesting process. Levin and Degani (2019) proposed a modular design of an agricultural robot structure by examining the phenomena of low reusability and narrow applicability of the harvesting robot structure, which has resulted in a large improvement in harvesting time and fruit-harvesting success rate. Wang et al. (2019) proposed an optimization method of harvesting posture to address the randomness of the citrus growth direction on stalks and designed an occluding end effector with a success rate of fruit stalk-shearing up to 89% and a harvesting success rate of the best posture up to 74%. Kurtser and Edan (2020) used a TSP approach to plan a work sequence and path of sensing and harvesting tasks for a bell pepper-harvesting robot and concluded that planning a series of tasks can reduce costs by 12%. These equipments and methods were only commissioned in the laboratory and not in a realistic agricultural environment (Kurtser and Edan, 2020).

Compared to single-arm robots, harvesting robots that use a two-armed operational strategy are more advantageous in grape trellises. The dual-arm robot extends up to 2.5 m and can cover all grape-growing areas of a standard trellis, and the harvesting efficiency is much higher than that of traditional robots. Zhao et al. (2016b) designed and tested a dual-arm frame equipped with two 3 DoF (degree of freedom) manipulators and two different types of end effectors used to pick tomatoes and exchanged the operator’s commands and displayed the state information of the robot. Ling et al. (2019) developed a dual-arm cooperative approach for a tomato-harvesting robot using a binocular vision sensor, and with vacuum cup grasping and wide-range cutting, the success rate of robotic harvesting reached 87.5%, while the harvesting cycle time was more than 30 s. Yu et al. (2021) used an autonomous humanoid robot for apple harvesting. It shows success rates of 82.5 and 72% for the apple recognition and harvesting functions, respectively; however, the apple-harvesting time is more than 30 s, and it has a rough structure and end effector. The authors concluded that although some progress has been made in the development of current grape-harvesting robots, further research is essential. Dual-arm harvesting robots can substantially improve operational efficiency, but there is still a lack of integrated harvesting robots in grape harvest production.

Grape clusters planted with trellises are mostly suspended on top of trellises, and the distance range from the cutting point of the fruit stalk to the top of the trellis is 30–100 mm, resulting in a small space for the upper limit activity of the robotic arm, which makes harvesting difficult and requires higher precision in identifying fruit clusters (Vrochidou et al., 2021). To accomplish efficient grape harvesting in standard trellis complex environments, our research group invented a dual-arm grape-harvesting robot for high standard trellis environments. Its harvesting structure used an RGB-D camera for the environmental field of view scanning and obtained the spatial information of grape-harvesting points and transmitted it to a dual robotic arm control system. The robot is a modular design. Facing different fruit and vegetable harvesting requirements, it only changes the structure of end effectors and adjusts the parameters of the vision recognition algorithm to quickly achieve a variety of fruit-harvesting tasks. Robotic harvesting operations through unmanned control have high harvesting quality and harvesting efficiency. They can significantly reduce the labor burden in grape harvesting and improve the efficiency of grape-harvesting operations (Zhao et al., 2016a; Ling et al., 2019; Seol et al., 2020).

Therefore, a dual-arm rapid grape-harvesting robot for the horizontal trellis was designed and analyzed in this article. This robot is integrated with a variety of sensors and actuators to enable unmanned operation processes. In section “Parameters of the horizontal trellis environment,” the horizontal trellis environment is introduced. In section “Overall structure of the dual-arm rapid harvesting robot,” the hardware and software architecture design of the robot for rapid harvesting is described. In section “Key technologies of dual arm rapid grape-harvesting robot,” we introduced the key technologies of the robot. First, we propose a “one eye-dual-arm” high-speed parallel harvesting strategy based on the structural parameters of the horizontal trellis. The position of the camera in relation to the two arms was also determined. Then end effectors and the vision algorithm were optimized for rapid recognition and harvesting process implementation. The combination of end effectors and a vision algorithm substantially improves the tolerance for errors. Finally, a dual-arm harvesting strategy based on depth values is proposed to achieve a harvesting sequence and the division of operation space by spatially symmetrical segmentation. For the area where the two arms are prone to collision, we established the danger area and safety area. In the danger area, the two arms will use an asynchronous master–slave dual-robotic arm anticollision harvesting strategy. In section “Experiments,” we present indoor accuracy experiments and field performance experiments. In section “Conclusion,” some conclusions are provided. Meanwhile, the existing work deficiencies and future research work are discussed.

## Materials and methods

### Parameters of the horizontal trellis environment

**FIGURE 1:**
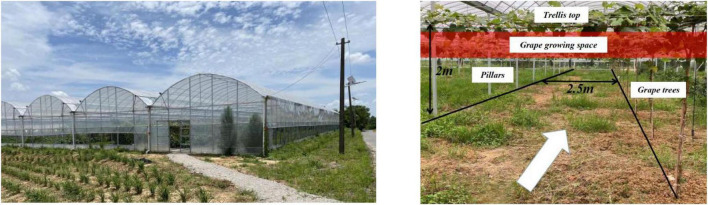
Vineyard in Jurong City.

The viticulture mode in horizontal trellises is the grape tree-planting method, in which the bottom of the trellis is supported by pillars, and the top is pulled by cross bars or lead wires to form a net-like shelf surface, and branches and vines grow on the shelf (Figure 1). A horizontal trellis has the advantages of ventilation, light penetration, easy branch management and high production. It has become one of the main modes of fresh grape cultivation.

The horizontal trellis is divided into two upper and lower layers by pulling a wire mesh at the top. The upper layer allows vine branches to grow and spread, confining a large number of branches and leaves to the upper area, while grapes grow by gravity and hang vertically downward, achieving separation between fruit, branches, and leaves. After several measurements, the height of grape clusters to the top of the trellis is usually 20–120 mm, and the height of the bottom of grape clusters to the ground is 1,700–1,900 mm.

Fresh grapes, as ready-to-eat fruit, need to meet the integrity and aesthetics of the bunches for later sale and eating, so there are higher operational standards for harvesting fresh grapes. Grape-harvesting methods with traditional trellises rely on manual hand harvesting, with one hand supporting grape bunches and the other hand shearing the fruit stem, which is harmful to health because of the long hours spent harvesting with a head-up posture. Based on this horizontal trellis, there is an urgent need to design an intelligent grape-harvesting robot for standard trellises to replace manual labor to complete tedious tasks. Most traditional fruit- and vegetable-harvesting robots use a single mechanical arm as the harvesting servo mechanism, resulting in extremely low single-cycle harvesting efficiency that is much lower than the manual operation efficiency and cannot meet the requirements of the grape industry.

We completed a study of horizontal trellises for fresh grapes in different vineyards in Jurong City, Jiangsu Province, China (119.25852°E, 31.88404°N). Standard grape trellises have many unique structural characteristics that harvesting robots need to adapt. In this particular working environment, the harvesting robot is required to meet the following design requirements.

(1) Based on the horizontal trellis structure and the vertical growth of grapes, the harvesting width, depth value of recognition range, walking step length and another factors as key parameters of this robot. Hand-eye combination configuration and the harvesting posture determine the range of this robot and the end-effector. For these special requirements from the environment structure, the analysis of robot construct with multi-parameters fusion becomes the central issue.
(2) The position of the camera relative to the robotic arms was determined to ensure that all grape-harvesting targets were fully integrated into the field of view in the camera and robotic arm harvesting range. The combined relationship between the camera and dual arms becomes the key point.
(3) The distribution of grape growth was random in the standard trellis. The harvesting robot needs to quickly identify grape targets and assign harvesting tasks to the two robotic arms to accomplish rapid and accurate visual servoing. Harvesting task assignment is an important prerequisite for visual servo.
(4) The robot needs a reasonable harvesting strategy. It can respond to unstructured environments in real time. It is a crucial technology for the robot to make the harvesting motion smoothly, accurately and at high speed.

### Overall structure of the dual-arm rapid harvesting robot

#### Hardware structure

Single-arm harvesting robots have some shortcomings, including a small operating width (1–1.5 m) and low harvesting efficiency (average of 25 s/cycle). Most robots rely on tractors for towing or rail transport (Kootstra et al., 2021). They are unable to navigate autonomously in response to agricultural environment changes. Therefore, it is extremely important to develop a robot with high harvesting performance, multisensor integration, and real-time sensing of environmental changes.

**FIGURE 2:**
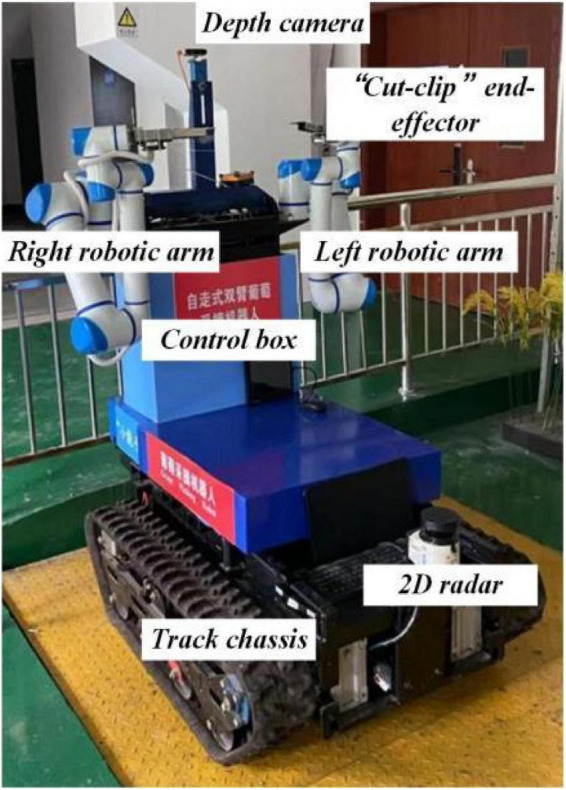
Dual-arm rapid grape-harvesting robot.

Figure 2 shows the developed dual-arm rapid grape-harvesting robot. Its structure includes a RealSense D435i depth camera, two 6 DoF robotic arms, and mobile tracked chassis. The RealSense D435i depth camera is mounted on top of the robot. This ensures that the camera obtains as much of the field of view as soon as possible. The RealSense D435i depth camera acquires the spatial coordinates of grape clusters by shooting a standard trellis environment. The two robotic arms are distributed with the camera mounting position as the center of symmetry. To ensure that the dual-arm working space covers the grape-growing space within the standard trellis, two robotic arms are mounted on both sides of the robot. Many sensors are integrated into the control box (Jetson Nano, STM32, robotic arm controllers). The camera and two robotic arms are mounted *via* steel to a mobile tracked chassis. To acquire a point cloud of grape trees in a reasonable view, a SICK 2D radar system is mounted on the front of the tracked chassis. Multiple electrical systems are integrated into a robot, and this robot can handle various requirements in a nonstructural agricultural environment.

#### System architecture

A Nvida Jetson Nano developer kit is used as the center of the decision system. Its small size and powerful computing power meet the needs of running programs in harvesting (GPU: 128-core NVIDIA Maxwall, CPU: Quad-CoreARM Cortex-A57 MPCore). RealSense D435i depth camera is used as the main sensor to obtain environmental information (RGB images 1,920 × 1,080 in resolution, depth images 1,280 × 720 in resolution, with a FOV of 69° × 42°). It is manufactured by Intel, United States. It is able to cover a wider area and reduce more blind spots. The robotic arms use Techsoft TB6-R5 (Techsoft, Shenzhen, China). TB6-R5 has a payload of 5 kg and repeatable position accuracy of up to ±0.05 mm. Each robotic arm has its own controllers, and controllers receive their respective harvesting tasks and control independently. Each arm is equipped with a cut-clip end effector. They hold grape clusters while cutting grape stems. The chassis is manufactured by Sangpu Agricultural Machinery Co., Changzhou, China. This robot uses a 2D radar (LMS-111, Sick, Germany) with TOF distance detection and enables accurate measurements in a complex field environment.

The dual-arm grape-harvesting robot consists of four main units: (1) a visual recognition system, (2) a decision system, (3) a servo harvesting system, and (4) a walking chassis system. The four units communicate with each other and work together to harvest the target fruit based on visual information.

**FIGURE 3:**
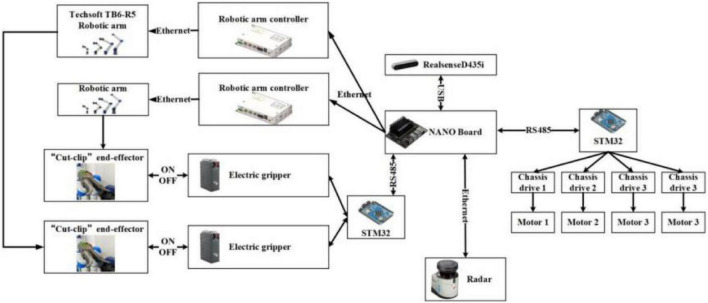
Hardware communication method.

To adapt to the field environment in agriculture, agricultural robotic systems are often required to have strong integration. Figure 3 illustrates the details of the control system for the whole robot. In the hardware section, Jetson Nano is responsible for key aspects such as image processing, motion information transmission, and communication between each hardware unit. RealSense D435i is connected to Jetson Nano *via* USB and sends the 3D information acquired to Jetson Nano in real time. These images are segmented, and the algorithm extracts contour information within ROS (robot operating system). The robotic arm (Techsoft, TB6-R5, CHN) communicates with the controller in real time *via* an EtherCAT bus. After the robotic arm moves to the target coordinate, it sends a signal to Jetson Nano. Jetson Nano sends control commands to STM32 through serial ports. STM32 controls the opening and closing of the electric gripper. When there are no harvesting targets in the camera field of view, the chassis moves forward some distance. Until the camera requires harvesting targets again, the chassis stops moving, and then, the next harvesting cycle begins.

**FIGURE 4:**
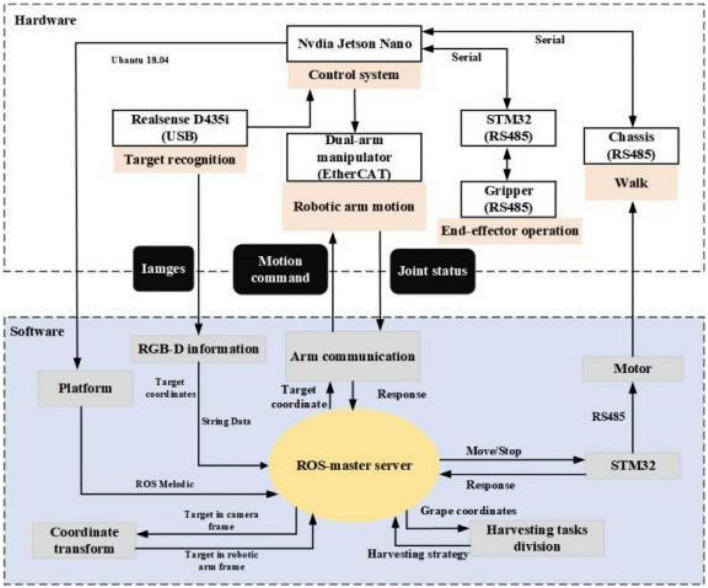
Control system of the dual-arm rapid harvesting robot.

In the software part, ROS is currently the most popular control system in robots. It is able to manage and transmit multiple sensor data. The data of the camera, robotic arms, grippers, and chassis are defined as nodes. These nodes subscribe to each other through topics for data delivery. The overall software component allows for a rapid response to agricultural environmental changes (Figure 4).

### Key technologies of dual arm rapid grape-harvesting robot

#### “One eye-dual hand” structure based on horizontal trellises

**FIGURE 5:**
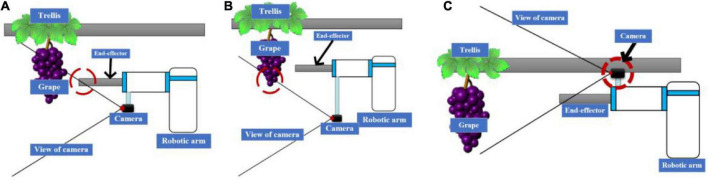
**(A)** Camera placed a short distance below. **(B)** Camera placed below at a distance. **(C)** Camera mounted above.

A hand-eye structure is the basis of robot vision servo control. At the same time, “eye-in-hand” usually requires a camera at the end of the arm. This results in a small camera field of view and cannot capture all the harvesting targets in the horizontal trellis. As shown in Figures 5A–C, three kinds of “eye-in-hand” structure occurs in different scenes.

**FIGURE 6:**
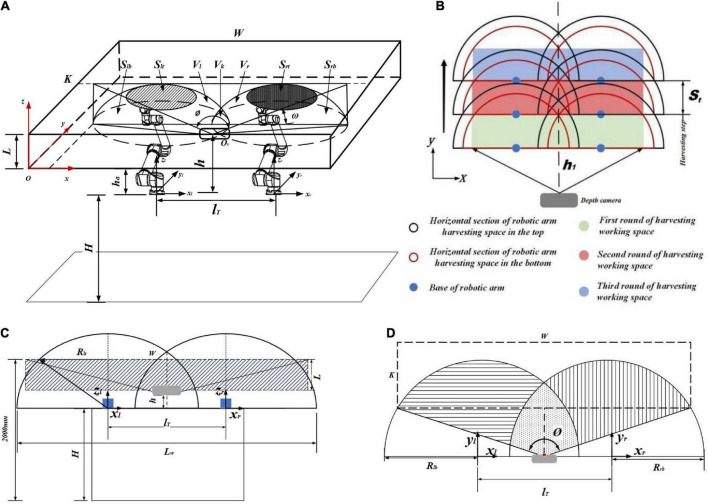
**(A)** Analysis of the “one eye-dual arm” working space and camera field of view. **(B)** Dual-arm working space and robot harvesting step. **(C)** Operating effect in the *XOZ* coordinate system. **(D)** Operating effect in the *XOY* coordinate system.

Therefore, the special “one eye-dual hand” structure is proposed. This structure ideally ensures full coverage of all grape clusters in grape-growing space under a horizontal trellis. The rational arrangement of the mounting position relationship between the two arms and camera becomes the core of the robot harvesting structure. To obtain as many grape clusters as possible, the camera field of view needs to match the dual-arm working space (Figure 6; Barth et al., 2016; Seyyedhasani et al., 2020; Chen et al., 2021).

A robotic arm working space is usually defined as a spherical space to simplify the problem in traditional research. However, the 6 DoFs robotic arm consists of motors and links. It does not have an ideal spherical shape of a robotic arm because of the difference in length and orientation of links. It looks more like a rugby ball in a working space. Therefore, it would be more reasonable to analyze robotic arm working space using the ellipsoidal spherical space equation.


(1) $$V=V_{l}+V_{r}-V_{k}$$

(2) $$V_{l}=V_{r}=∭\left(\frac{x^{2}}{b^{2}}+\frac{y^{2}}{a^{2}}+\frac{z^{2}}{c^{2}}\right)dydxdz$$

As shown in Figure 6A, the left robotic arm working space ***V*****_*l*_** and right robotic arm working space ***V*****_*r*_** are added and subtracted from the overlapping space ***V*****_*k*_**, which is the actual working space of the two arms ***V***.


(3) $$X=\frac{x}{b},Y=\frac{y}{a},Z=\frac{z}{c}$$

(4) $$V_{l}=V_{r}=a\,b\,c\,∭\left(X^{2}+Y^{2}+Z^{2}\right)dXdYdZ=\int_{0}^{L}d\,z\,\iint f\,\left(X,Y,Z\right)dXdY$$

(5) $$l_{k}=\frac{4\,a-L_{w}}{2}$$

(6) $$V_{k}=2\,\int_{4\,a-L_{w}}^{a}dx\,\iint f\,\left(X,Y,Z\right)\,dYdZ$$

(7) $$V=2\,\int_{0}^{L}dz\,\iint f\,\left(X,Y,Z\right)\,dXdY-2\,\int_{4\,a-L_{w}}^{a}dx\iint f\,\left(X,Y,Z\right)\,dYdZ$$

where ***V*** is the overlapping part of the dual-arm working space and grape-growing space, ***W*** is the grape-growing space length, ***K*** is the grape-growing space width, ***L*** is the grape-growing space height, **l**_*T*_ is the dual-arm mounting horizontal spacing, ***H*** is the height of the arm from the ground, **h**_*a*_ is the height of the arm from the grape-growing space, **S**_lt_ is the top area of the left arm working space and grape-growing space, **S**_lb_ is the bottom area of the left arm working space and grape-growing space, **S**_**r***t*_ is the top area of the right arm working space and grape-growing space, **S**_rb_ is the bottom area of the right arm working space and grape-growing space, and **L**_*w*_ is the working width of the two arms. **O**_*v*_ is the camera mounting position, ***h*** is the camera mounting height.

The area where the dual-arm workspace and camera field of view overlap is the area of harvesting that the robot can identify and harvest. Unreasonable arrangement of robot harvesting steps can effectively reduce the harvesting efficiency in grape-growing space and increase the number of missed grape targets. As shown in Figure 6B, the width of the camera field of view needs to be greater than the width of harvesting space ***W***. The camera field of view takes the camera as the vertex. The directions of the FOV angle are extended. The shape of view is similar to a quadrilateral cone. By calculating the camera FOV angle, the camera field of view equation is derived. Threshold segmentation of the camera field of view effectively limits the range of the camera shot and filters interference.

From Figures 6B–D, we established the camera field-of-view equations. Relevant parameter constraints were established based on the horizontal trellis, camera field-of-view range, and dual-arm working space.

Camera field of view space:


(8) $$\pm \frac{x_{o}}{t\,a\,n\,\frac{\emptyset }{2}}\pm \frac{z_{o}}{t\,a\,n\,\frac{\omega }{2}}-2\,y_{o}=0$$

where ∅ is the camera shooting horizontal field-of-view angle, ω is the camera shooting vertical field-of-view angle, (x_o_,y_o_,z_o_) is the coordinate of the target point.


(9) $$\left\{ \begin{array}{c} 200\,m\,m\leq d_{v\,i\,e\,w}\leq h_{1}\,S_{t}\\ l_{r}+R_{l\,b}+R_{r\,b}\geq W\\ H+h+d_{h\,e\,i\,g\,h\,t}\geq 2000\,m\,m \end{array}\right.$$

where *d*_view_ is the camera depth threshold range, *d*_height_ is the height of the camera field, *S*_*t*_ is the harvesting step of the robot, *h*_1_ is the distance between the camera and robotic arms in the *y* direction, **l**_*r*_ is the mounting distance between dual arms, *R*_lb_ is the minimum working width of the left robotic arm in grape growing space, **R**_*rb*_ is the minimum working width of the right robotic arm in grape growing space.

By combining the characteristics of the horizontal trellis, camera field of view, and dual-arm working space, we obtain a reasonable installation position relationship between the camera and two arms:


(10) $$\left\{ \begin{array}{c} \begin{array}{c} S_{t}=800\,m\,m\\ H=1400\,m\,m \end{array}\\ h=300\,m\,m\\ \begin{array}{c} l_{r}=1100\,m\,m\\ h_{1}=250\,m\,m \end{array} \end{array}\right.$$

**FIGURE 7:**
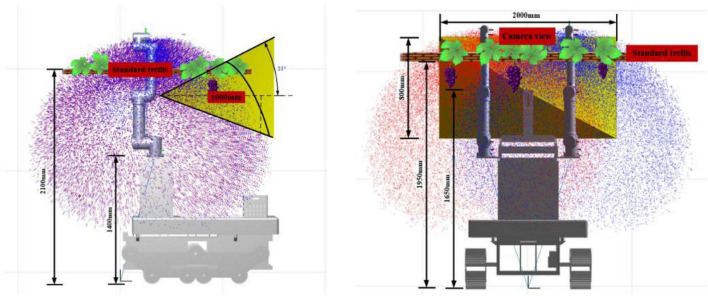
Operating space point cloud of the dual-arm harvesting robot.

As shown in Figure 7, after MATLAB with Solidworks simulation, the “one eye-dual arm” structure ensures that the ends of the robot arm have sufficient space to move the trellis boundary so that the ends of the robot arm can reach the farthest end of the horizontal scaffolding in a flexible posture to complete harvesting operations, and the robot can be made in a harmonious proportion similar to the human form configuration without a lack of design aesthetics.

#### Large error tolerance of the “hand-eye” combination

(1) Rapid identification of multiple targets in one image.

The images of grapes inside a horizontal trellis obtained by RealSense D435i often exhibit multiple clusters of grapes. If each bunch of grapes needs to be identified once by the camera, it would greatly increase the harvesting time. To achieve rapid harvesting of multiple bunches of grapes in a horizontal trellis, it is necessary to achieve rapid identification of multiple bunches of grapes within an image.

**FIGURE 8:**
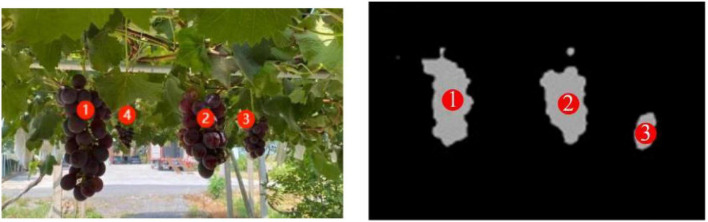
Identification of multiple bunches of grape clusters within one image.

Multiple bunches of grape bunches were often targeted in the images of grapes inside the horizontal trellis obtained by RealSense D435i. The camera directly acquires the depth values of all grapes in the image. According to Figure 8, we can calculate the camera depth threshold range as follows:


(11) $$100\,m\,m<d<S_{t}+h_{1}$$

When grape clusters are less than 100 mm from the camera, the camera cannot focus on the grapes. When grape clusters exceed the depth threshold, grape targets are beyond the working space of the two arms. As shown in Figure 8, four grape clusters were present in the image. The fourth grape cluster was cleared as background because the depth value exceeded the depth threshold. The other three grape clusters were harvested based on the depth value from smallest to largest.

(2) Fuzzy prediction of grape stem-cutting points based on grape contours.

**FIGURE 9:**
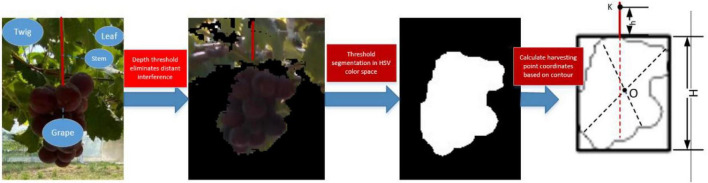
Calculation of the stem-cutting point.

Grape leaves, stems that are non-grapes, and grape clusters in trellises can interfere with the target stem identification in traditional algorithms. However, this fuzzy algorithm does not rely on the precise identification of grape stems. The algorithm constructs an external rectangle of grapes by HSV thresholds acquiring their geometric contours. The center of the external rectangle is the center of the grape profile in this algorithm and moves upward to speculate the coordinates of grape stems. When there is a small amount of cover in grape clusters, this algorithm can still quickly determine the inference of grape stem coordinates (Figure 9).

Grape contours were bounded to obtain parameter spike length and width values, and the center of grape coordinates *O (x*_0_, *y*_0_, *z*_0_) was calculated based on the distribution area. *z*_0_ is the depth value of the center of the grape coordinate from the camera and can be obtained directly through the depth camera. The spatial coordinates of the grape-harvesting point *K (x_*k*_, y_*k*_, z_*k*_*) are calculated as follows:


(12) $$\left\{ \begin{array}{c} {\text{x}}_{k}=x_{0}\\ y_{k}=y_{0}+\frac{H}{2}+h\\ z_{k}=z_{0} \end{array}\right.$$

(3) “Cut-clip” end effector for grape horizontal trellises.

Traditional finger end effectors often damage grapes at the finger end during the grape harvesting process. For Kyoho grapes, the stalks can reach 15 mm in diameter, and the weight of a single cluster can reach 400 g. RealSense D435i extracts the geometric contours of grape clusters to infer the calculation of grape stem-cutting points with visual recognition errors. Due to hand-eye calibration and coordinate conversion, the movement of the robotic arm has some motion errors. Both of these errors are generated by the design principle and algorithm. They are difficult to reduce or minimize. The end effector must grip the whole grape cluster when cutting the stem. It needs to be transported from the standard trellis to the fruit box smoothly to ensure no damage.

**FIGURE 10:**
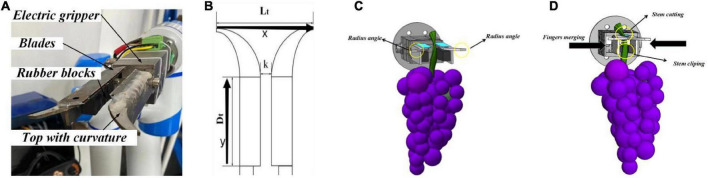
**(A)** Construction of the end effector; **(B)** error tolerance in the end effector; **(C)** end effector opening; and **(D)** end effector closing.

Facing these requirements in grape harvesting, our research group has designed an end effector for rapid grape harvesting. The finger of the end effector is designed with certain curved angles. When the end effector begins to harvest, the finger with curved angles can reduce the negative effect of visual recognition errors and arm motion errors (Figures 10A,B). This structure enhances error tolerance in the *x* and *y* directions. It turns a harvesting point into a harvesting range.

Motors are used to control the fingers to open and close. Three sets of blades are mounted on the fingers. When the fingers are closed, the blades finish cutting the grape stems. At the same time, the lower part of the fingers is fitted with a rubber block to hold grape clusters to cut the stems (Figure 10C). The end effector is simple in structure and only requires ± signals to complete the control process (Figure 10D). It enables the integration of cut-clip multitasking in rapid grape-harvesting tasks and significantly improves the harvesting efficiency and success rate.

(4) Error tolerant combination of the end effector and vision algorithms.

Nonstructural features exist within the horizontal trellis. Images contain not only grape clusters but also branches, leaves, the trellis, and another environment. It is a challenge to quickly acquire grape-harvesting points from complex backgrounds. When grape stems are obscured, interlaced, or overlapped, the stem-cutting point error is large. This leads to chaotic robotic arm movements, harvesting failures, and serious collision problems.

**FIGURE 11:**
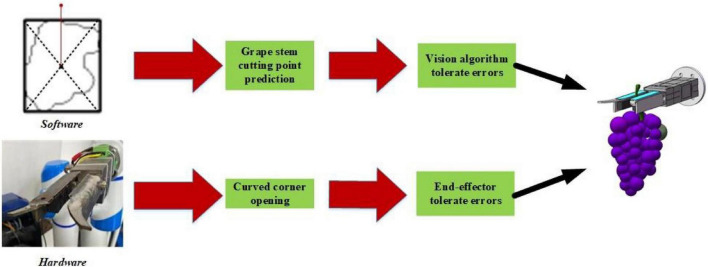
Error tolerance combinations of the end effector and vision algorithms.

Faced with the special grape-harvesting requirement, our group obtained the coordinates of the center of the external rectangle based on the grape geometric profile and thus achieved the vertical upward prediction of grape stem-cutting points. By using the external rectangle of the grape cluster to predict stem-cutting points, even if grapes are partially obscured by the outline, the stem-cutting points can be predicted by the external rectangle with little error (Figure 11).

By the fuzzy prediction method for the grape stem-cutting points, grape clusters in horizontal trellises can be quickly recognized. When grape clusters were partially shaded, the external rectangle of grape clusters did not change significantly. Stem-cutting points were predicted by the external rectangle of grapes to reduce the errors of grape clusters in images. When the fingers of the end effectors have curved angles, the error of the stem-cutting points in the horizontal direction can be enhanced. As shown in Figure 10B, the design of the end effector produces some horizontal error tolerance degree ***L*****_*t*_** and depth error tolerance degree ***D*****_*t*_**. It expands the original visually identified point into an area and improves the harvesting success rate.

It guides the stem to the area where the blade will cut. By the end effector mechanism, the point of the grape stem can be converted into an area range. This tolerable error method that combines software and hardware has significantly increased the success rate and harvesting efficiency of grapes in horizontal trellises.

#### Dual-arm harvesting strategies in the horizontal trellis

(1) Based on depth value “symmetric space segmentation” harvesting sequence.

**FIGURE 12:**
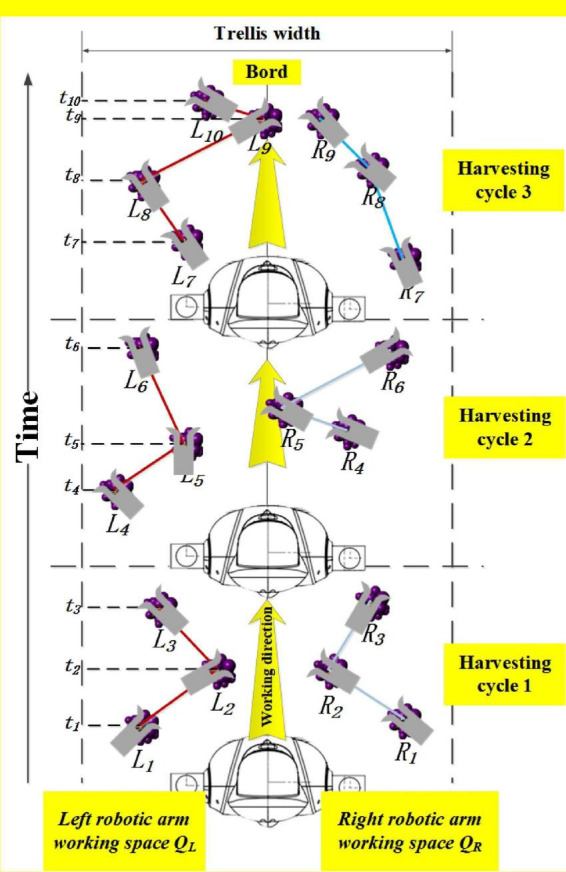
Dual-arm harvesting sequence in a horizontal trellis.

Dual arms are not just a superposition of the operational efficiency of two robotic arms. The disorderly and random distribution of grapes on horizontal trellises means that the harvesting sequence and path for robotic arm harvesting operations need to be planned (Takano et al., 2019). Grape clusters captured by the camera view become harvesting targets, and the center axis plane of the camera field is used as the operation space segmentation reference plane. We divided the camera view into left working space and right working space based on the “symmetric space segmentation” method. Finally, the coordinating information of target grapes is sorted based on the depth values and transferred to the Cartesian coordinate system. When the target grape coordinate *x* < 0, the harvesting task is divided into the left arm workspace, and when *x* > 0, it is assigned to the right arm workspace. This ensures independent parallel operation between two robotic arms without interference and joint collision (Figure 12).

From Figure 12, multiple bunches of grapes L_1_−L_3_ and R_1_−R_3_ were found in the field of view of the camera in harvesting cycle 1. Dual arms harvest the target grape in their respective areas until all of them are harvested. When there are no grape targets in the camera field of view, the chassis will automatically run into harvesting cycle 2 and will harvest grape clusters L_4_–L_6_ and R_4_−R_6_.

(2) Danger and safety areas for dual-arm operation.

**FIGURE 13:**
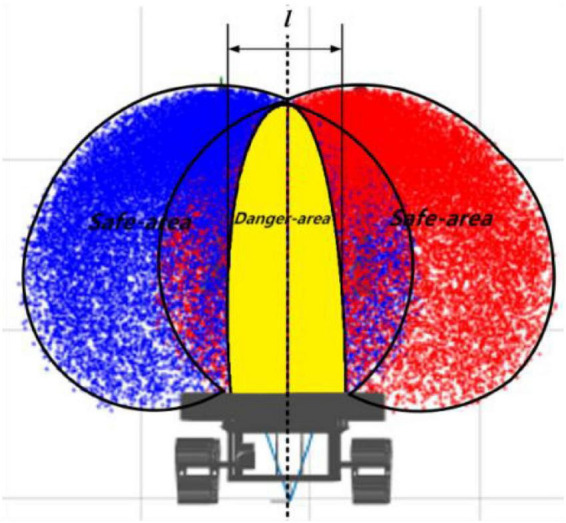
Danger area and safe area in dual-arm operating space.

Robotic arms are used as electrical devices with independent control centers. Dual arms may be prone to collision and even serious damage. Therefore, we defined a dual-arm operating space and established a danger area and safe area in the working space. The fixed area in yellow shown in Figure 13 can be named the danger area. This means that we need to perform two scenario analyses:

(1) When grape targets are present in the safe area, the two arms do not collide. Therefore, the two arms can harvest independently and speedily without the need to restrict harvesting movement. Therefore, in this case, there is no need to change the strategy.
(2) If multiple bunches of grapes are present in the danger area, how to ensure that robotic arms can still complete the rapid harvesting process without collision must be determined.
(3) Asynchronous master–slave dual-robotic arm anticollision harvesting strategy in the danger area.

The danger area is a smaller part of the operating space of the dual arms. When multiple bunches of grape clusters are growing in the danger area, the movement of the two arms requires very careful planning. Otherwise, arm joints or end effectors are prone to collision. Our group proposes an asynchronous master–slave dual-robotic arm anticollision harvesting strategy in the danger area. This strategy is based on the conditional judgment of the grape cluster distribution location, as shown in Table 1.

**TABLE 1:** Different strategies for grape targets in different areas.

Scene	Use this strategy	Harvesting path
The two arms are out of the danger area	×	There is no risk of collision of the two arms, and independent and rapid harvesting can be achieved.
One arm in the danger area, the other arm in the safety area	√	The one arm in the danger area is treated as the master arm and has the priority of harvesting. The other arm needs to wait to complete its harvesting action before it starts moving.
The two arms are in the danger area	√	When grape targets are in the danger area, the harvesting priority of the arm needs to be determined based on the grape-harvesting order. The robotic arm with the priority will become the master arm. It will enter the danger area to harvest first.

**FIGURE 14:**
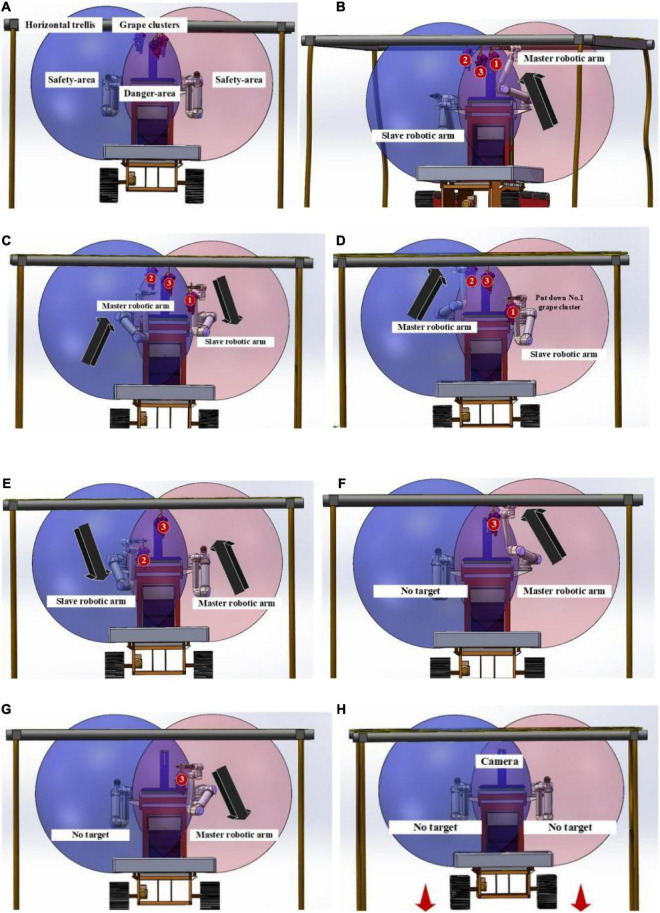
Asynchronous master–slave dual-robotic arm anticollision harvesting strategy in the danger area.

The danger area occupies only a small part of the working space of the two arms. Therefore, the probability of this strategy being employed by two arms tends to be small, which does ensure the safety of robotic arms in harvesting work. As shown in Figure 14A, when multiple bunches of grapes are present in the danger area, the dual-arm strategy will be used for safe and rapid harvesting.

From Figure 14B, three bunches of grapes are in the danger area. Grape-harvesting tasks are divided into the left arm for bunches 1 and 3 and the right arm for bunch 2. At this time, the left arm acts as the master robotic arm, and it has the priority of harvesting the danger area. The right arm is a slave arm, and it needs to wait for the signal that the master arm has finished harvesting tasks. Then, it starts its harvesting mission.

From Figure 14C, there was a shift in the master–slave relationship between the two arms. The left arm moved down and out of the danger area. It was transformed from a master robot to a slave arm. Meanwhile, the right arm moved toward bunch 2 of the grape cluster in the danger area. It had priority access to the danger area for harvesting. The two arms entered the danger area for harvesting at different times. There is a time gap between the two arms in harvesting work.

Figures 14D–G show that converting the master–slave relationship between the two arms can ensure that the two arms work independently and smoothly in the danger area. When the robot adopts this strategy, it can reasonably use the time difference and robotic arm movement motion position in space. The high-speed harvesting work of the two arms in the danger area is an extremely difficult and complex task. In agricultural non-structural environments, a reasonable motion strategy for two arms often leads to great safety and efficiency improvements in the robot.

As shown in Figure 14H, if there is no harvesting target in the camera field of view, the robot will move some distance forward. A new harvesting cycle will start.

## Experiments

### Materials and methods

**FIGURE 15:**
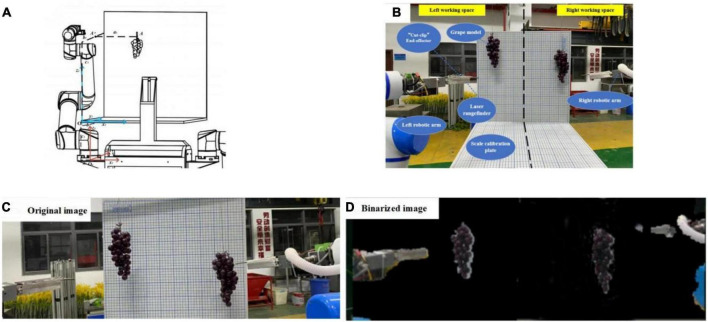
Figures of the indoor experiment: **(A)** harvesting point coordinate transformation, **(B)** indoor experimental schematic, **(C)** experimental original image, and **(D)** binarized image of grapes.

To verify the accuracy of large tolerance of the “hand-eye” combination and robot performance, both trellis and lab experiments were carried out:

(1) To test the accuracy and efficiency of the robotic arm in harvesting operations, we acquired the experimental errors in the harvesting process. An experimental platform was designed and built to finish grape cluster harvesting in a room. The grape-harvesting accuracy experimental platform is designed and produced, and two scale plates (0.5 m × 0.5 m) with a 2 mm grid size are combined and matched to form a coordinate experimental platform in Cartesian coordinates. The accuracy and performance of large error tolerance of the “hand-eye” combination were verified by every 30 harvesting experiments with shading grape clusters to different degrees (0–5, 6–20, and 21–40%). This platform can measure the coordinates of grape stem cutting point A by converting the robotic arm base coordinate system O_1_ to the platform coordinate system O_2_, and compare it with the visual recognition point and robotic arm motion point to derive a visual recognition accuracy error(mm) and arm positioning accuracy error(mm). Meanwhile, it needs to record harvesting time(s) and harvesting success rate (Figures 15A–D).

(2) Trellis performance experiments.

**FIGURE 16:**
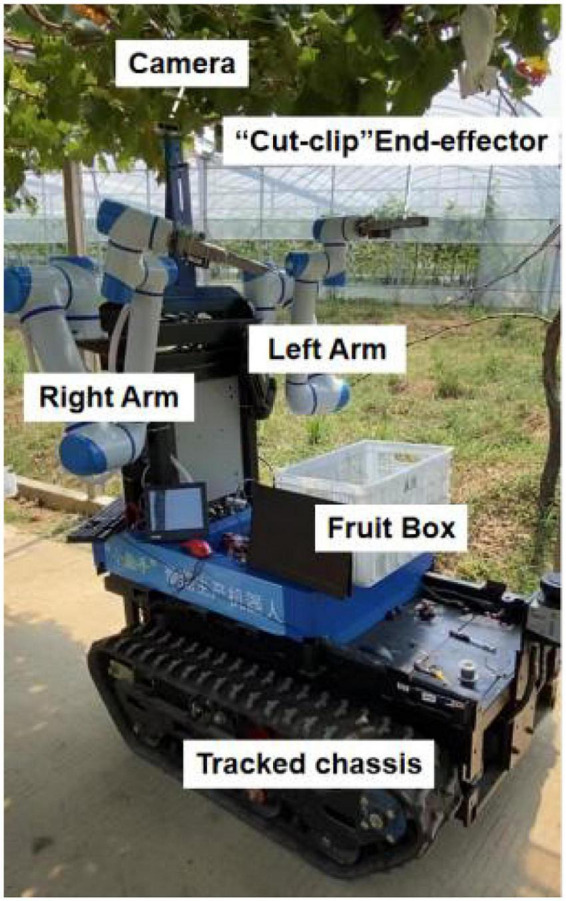
Dual-arm robot performing grape-harvesting operations.

The experiments were conducted in September 2021 at the ErYa Vineyard in Jurong City, Jiangsu Province, China, where grapes were grown with a horizontal trellis type of cultivation. In this vineyard, grapes grew in good conditions, with most of the clusters hanging vertically below the trellis. The grape variety was Kyoho, which has large clusters, large grains, and purple–black fruits at maturity and is the main variety grown in grape production in China. With a horizontal trellis height of 2.0 m, a width of 2.5 m, and a trellis length of 30 m, this robot can meet the full range of coverage for harvesting in a single cycle inter row grape environment. There are no other obstacles around the grape-harvesting area, which can ensure that no exogenous emergency stopping occurs during the operation of the robot. The robotic harvesting process was captured in real time by the camera, recording the recognition success rate, harvesting success rate, and harvesting time of one grape cluster (Figure 16).

### Results and discussion

(1) Lab experiments.

From Table 2, we know that the maximum visual recognition accuracy errors in the *x*, *y*, and *z* directions from the robotic arm base were 15.147, 13.689, and 16.330 mm, respectively, as the degree of obscuration of the grape bunches by the leaves increased, thus showing that the integrity of grape bunches’ contours accounted for a great deal of the impact on the visual recognition accuracy of the camera. The motion positioning errors of the left and right robotic arms were 2.885, 3.972, and 2.715 mm and 2.471, 3.289, and 3.775 mm, respectively, indicating that these robotic arms were well positioned and could support end effectors in reaching the grape-harvesting point accurately. The above errors were adjusted by the structure and design of the end effector, which can be applied to the grape-harvesting accuracy requirements under the operating conditions of a horizontal trellis. The average single-cycle completion time is 8.45 s. To explore and optimize the visual recognition capability of this robot, harvest failure tests were analyzed.

**TABLE 2:** Accuracy experiment results.

Degree of leaf shade (%)	Visual recognition accuracy error (mm)	Arm positioning accuracy error (left arm) (mm)	Arm positioning accuracy error (right arm) (mm)	Grape harvesting time (s)	Success rate (%)
0–5	(10.899, 8.552, 6.337)	(2.098, 3.003, 3.539)	(2.964, 2.363, 2.086)	8.47	93.3
6–20	(11.502, 10.141, 12.639)	(3.497, 3.551, 2.314)	(3.443, 3.605, 5.203)	8.23	86.7
21–40	(15.147, 13.689, 16.330)	(3.060, 5.363, 2.292)	(3.414, 3.901, 4.036)	8.66	73.3

From harvesting failure tests, the binarized images show grape cluster contours, and the grapes not obscured by the leaves are easily obtained as complete contours, allowing accurate calculation of the stem-cutting location. However, the grape area shrinks with increasing leaf occlusion resulting in many deviations in the center of the grape contour and the stem-cutting point coordinates. This affected the success rate of subsequent harvesting by robotic arms. After subsequent iterations and changes in test conditions, the factors affecting this phenomenon were identified.

• Uneven light distribution.

Influenced by the sunlight irradiation direction and grape growth contour, the images captured by the camera were incomplete, with abnormalities such as mutilation, deformation, and overlap of grape clusters within the images, resulting in deviations in the generated grape stem-harvesting points. However, in a normal horizontal trellis, grape leaves and branches grow at the top of the trellis, and the sunlight intensity generally does not interfere greatly with camera recognition.

• Shaking of the grape model.

The selected grape model was made of plastic, with low weight and weak resistance to external interference, resulting in slight shaking during photography. Before the trellis performance test, lighting was installed on the head of this robot to reduce the interference of natural light on camera recognition. The grapes planted in the trellis were hung vertically from the top, and the average weight of each grape cluster was close to 400 g. Thus, they were highly resistant to external interference and therefore only slightly swayed, with minimal effect on camera recognition.

(2) Trellis experiments.

This grape-harvesting robot advanced to the horizontal trellis and started harvesting above this trellis with the “sequential mirroring” strategy based on the depth information. The grape damage rate is the mass of grape clusters from falls, breaks, and bruises as a percentage of the mass of all harvested grape clusters. The number of harvesting successes, single-cycle dual-arm harvesting time, and grape damage rate was used as the main indicators to measure the quality of the dual-arm grape-harvesting robot in the tests (Table 3).

**TABLE 3:** Trellis performance experiment results.

Grape cluster ID/number	Successful visual recognition	Successful harvest	One grape cluster harvesting time/s	Damaged grains/number	Grape damage rate
1	√	√	8.14	0	0
2	√	√	8.76	0	0
3	√	√	8.60	0	0
4	√	√	8.93	0	0
5	√	√	9.43	0	0
6	×	×	–		
7	√	√	8.72	0	0
8	√	√	8.10	2	4.39%
9	×	×	–		
10	√	√	8.98	0	
11	√	√	9.25	1	2.02%
12	√	√	8.78	0	0
13	√	√	9.34	0	0
14	√	√	8.64	0	0
15	√	√	8.19	0	0
Average	86.7%	86.7%	8.76	0.23

• Results of visual identification.

The visual images of the dual-arm grape-harvesting robot show the fruit shape contour, segmented depth threshold, and grape target binarization image. The vision system calculates the image center of the grape-based on the binarization recognition image and derives the Cartesian spatial coordinate information of the grape stem-harvesting points. Among the 15 sets of experiments, 13 sets of experiments were completed. The fusion of depth information and color information determines the harvesting order arrangement, and the visual localization accuracy reaches 86.7% without neural network training, which can realize fast localization recognition in normal agricultural harvesting work.

• Continuous grape-harvesting test.

**FIGURE 17:**
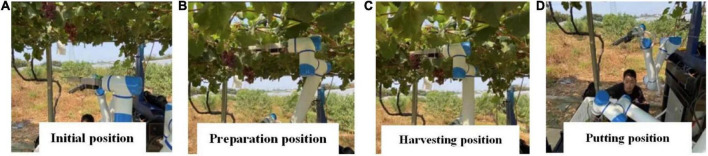
Robotic arm harvesting postures: **(A)** initial position; **(B)** harvesting preparation; position **(C)** harvesting position; and **(D)** putting position.

By analyzing the dynamics of each harvesting process and stem separation points of robotic arms, we analyzed the displacement change relationship between the stem and grapes during the grape-harvesting process and verified the single journey continuous harvesting method from the initial position, harvesting preparation point, harvesting point, and grape-putting point. The data in Table 3 show that the success rate of harvesting is 86.7%, and the main reason for the failure is the small size of the grape, which affects the correct conversion of the final harvesting coordinates. After 13 successful harvesting tests, the average harvesting time of one grape cluster is 8.76 s. The operating speed of the robotic arm was only set to 40% of the maximum speed of joint motion, and the single-cycle operating efficiency was still greatly improved after the subsequent structural stabilization of the robot (Figures 17A–D).

And we compared it with some currently used fruit and vegetables harvesting dual arms robots as shown in Table 4.

**TABLE 4:** Comparison between dual arms robots.

References	Products	Harvesting type	Harvesting success rate (%)	Harvesting efficiency (amount/hour)	Scenes
Arad et al., 2020	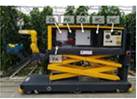	Sweet pepper	61	24	Greenhouse
Yu et al., 2021	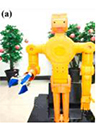	Apple	72	14.6	Indoor
Ling et al., 2019	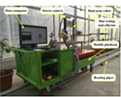	Tomato	87.5	30	Greenhouse
SepúLveda et al., 2020	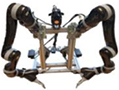	Aubergine	91.67	26	Indoor
Yoshida et al., 2022	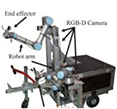	Apple	/	10	Field
This research	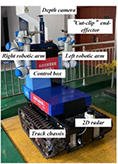	Grape	86.7	8.76	Field

By comparing these advanced harvesting dual arms robots, our robot has a faster harvesting efficiency, and reliable harvesting success rate and can be adapted to the complex vineyard.

## Conclusion

As a multipurpose fruit that easily falls off or break, how to achieve rapid and undamaged harvesting of grapes has become an urgent problem for the current grape industry worldwide. In this study, a dual-arm grape-harvesting robot is developed based on grape-harvesting demand in the special growing environment of horizontal trellises. This robot accomplishes the fusion and extraction of spatial multitarget information by a single depth camera and simplifies the calculation of 3D graphic information into spatial point coordinates. A “one eye-to-dual hands” vision servo system is built, and a single RGB-D camera is used to divide the field of view into equal tasks for two robotic arms, to locate multiple grape targets quickly and continuously, and transmit the spatial information of grape harvesting to the corresponding two robotic arms based on the corresponding spatial growth position distribution. The whole process of rapidly harvesting grapes was completed by transforming visual information and digital information into robotic machine signals.

To simulate the real environment of grape harvesting in the horizontal trellis, 30 sets of positioning accuracy tests were conducted with different degrees of leaf shading. Without neural network training, when the degree of leaf shading was 0–5%, the harvesting success rate was 93.3%, and one grape cluster harvesting time was 8.47 s. When the degree of leaf shading was 6–20%, the harvesting success rate was 86.7%, and one grape cluster harvesting time was 8.23 s. When the degree of leaf shading was 21–40%, the harvesting success rate was 73.3%, and one grape cluster harvesting time was 8.66 s, which met the requirements of rapid location identification and low-loss harvesting of grape clusters in a real horizontal trellis environment. After the trellis performance harvesting test, out of the 15 sets of experiments, 13 sets of experiments were completed with accurate identification, the visual positioning accuracy reached 86.7%, and the average harvesting time of one grape cluster was 8.76 s without neural network training, so fast positioning identification and rapid low-loss harvesting of grape clusters were achieved in a real horizontal scaffolding environment.

In the next step, because grape harvesting is still not faster than human harvesting, we will continue to work on optimizing all aspects of the robotic arm harvesting motion process. At the same time, we will conduct research on the minimization of robotic arm motion paths in nonstructural environments. The work of two arms in grape harvesting still holds great promise for research. All the technical details will be reported in the next study.

## Data availability statement

The raw data supporting the conclusions of this article will be made available by the authors, without undue reservation.

## Author contributions

YJ and JL: conceptualization and validation. YJ, JL, and JW: methodology. YJ: software. JW, WL, HS, and YP: formal analysis. YJ, WL, and YP: investigation. All authors contributed to the article and approved the submitted version.
